# Physical activity and health-related quality of life in chronic low back pain patients: a cross-sectional study

**DOI:** 10.1186/s12891-015-0527-0

**Published:** 2015-03-19

**Authors:** Andrea Schaller, Lea Dejonghe, Burkhard Haastert, Ingo Froboese

**Affiliations:** Institute of Health Promotion and Clinical Movement Science, German Sport University Cologne, Am Sportpark Muengersdorf 6, 50933 Cologne, Germany; mediStatistica, Lambertusweg 1b, 58809 Neuenrade, Germany

**Keywords:** Physical activity, Chronic low back pain, Health Related Quality of Life, Rehabilitation

## Abstract

**Background:**

The aim of the present study was to identify the relationship of physical activity (PA) and Health-Related Quality of Life (HRQoL) in patients suffering from low back pain (LBP).

**Methods:**

The present evaluation was conducted as a cross-sectional study based on baseline data of an randomized controlled trial on the effectiveness of an intervention promoting PA. Patients answered a questionnaire on domain specific PA (GPAQ) and HRQoL (EQ-5D-5 L). Furthermore, sociodemographic and indication-specific variables as well as work-related aspects were assessed. Associations of PA and HRQoL were estimated by means of regression analysis: one regression model only included domain specific PA (model 1) and a second regression model additionally included further variables (model 2).

**Results:**

412 patients completed the questionnaire. Model 1 showed opposed effects of workplace and leisure time PA: while workplace PA showed a negative association (β = −0.064; p = 0.04), a positive association of leisure time PA could be proved (β = 0.068; p = 0.01). Model 2 showed that only the variables “current work ability” (β = −0.030; p < 0.01) and “intensity of pain” (β = 0.104; p < 0.01) significantly contributed to explain the variance in HRQoL (model 2).

**Conclusions:**

The present results indicate the necessity of a differentiation of workplace and leisure time PA in the context of assessing health-enhancing effects of PA in LBP patients. In the context of HRQoL it must be assumed that the relevance of PA might be overestimated. Further research should be performed on predictors of HRQoL and thereby particular attention should be paid on the patients’ work-related and indication-specific aspects.

## Background

Physical activity (PA) is widely recognized as an important health-related lifestyle factor. The social, psychosocial and also biological health benefits of PA and exercise are well established and there is clear scientific evidence that moderate PA on a regular basis can reduce the risk of the morbidity of preventable non-communicable diseases (NCDs) [1,2]. Overall, lack of PA is mentioned as one of the four behavioral risk factors causing NCDs [3,4]. Also the benefit of regular PA in primary and secondary prevention as well as in the rehabilitative treatment of several NCDs is scientifically recognized. Observational and interventional studies proved positive effects of PA for diabetes, hypertension, cancer (esp. breast and colon cancer), osteoporosis, cardiovascular disease, obesity and depression [5,6]. World Health Organisation (WHO) guidelines addressing healthy adults and adults with NCDs not related to mobility (e.g., such as hypertension or diabetes) recommend at least 150 minutes of moderate-intensity or 75 minutes of vigorous-intensity aerobic PA throughout the week or an equivalent combination of moderate- and vigorous-intensity activity. Independent of intensity, aerobic activity should be performed in bouts of at least 10 minutes duration [7]. These recommendations relate not only to sports, but also to other leisure activities, work and transport activity [7].

Besides morbidity and mortality, patient-oriented outcomes, such as Health-Related Quality of Life (HRQoL), are of utmost importance in rehabilitation and health services research [8]. While quality of life is defined as a broad concept that incorporates all aspects of an individual’s existence, HRQoL is considered to be a subset relating only to the health domain of that existence [9]. For developing patient-oriented treatments it is of utmost importance to understand what influences HRQoL. In this context, the associations of lifestyle factors, such as PA behavior, and HRQoL need to be examined to identify target-group specific approaches to promote HRQoL. Concerning the influence of PA on HRQoL, a systematic review showed a positive association in general population [10] and PA is considered as a lifestyle factor having the potential to increase the quality of life [2]. Compared to healthy persons, a lower HRQoL of patients suffering from NCDs could be demonstrated [11,12] but it also could be shown that physically active persons in this population group tend to improved HRQL [13].

Low back pain (LBP) is not only one of the leading causes of pain and disability but also of a costly burden on the healthcare budget as LBP leads to a frequent demand for medical services [14,15]. The lifetime prevalence of LBP is estimated 74% to 85% and almost 20% of the population is suffering from either severe or disabling back pain [16,17]. Despite the relevance of LBP patients in health services research, the associations of lifestyle factors on HRQoL in this patient group are rarely examined. PA is ascribed a potential role in the prevention of chronic LBP and therefore can be considered as an essential lifestyle in LBP patients [18-20]. Furthermore, the relevance of PA in the context of LBP is ascribed to its potential to reduce potential risk factors of LBP, e.g., obesity [21].

A better understanding of how PA is associated to HRQoL contributes to a comprehensive understanding of HRQoL and also to the development of target-group specific approaches to increase HRQoL in LBP patients. Therefore, the present paper aims to 1) explore the association between domain specific PA and HRQoL and 2) identify variables associated with HRQoL in LBP patients.

## Methods

### Subjects and study design

Data were collected as part of a randomized controlled trial (RCT), evaluating the effectiveness of an intervention on physical activity promotion (German Clinical Trials Register (DRKS)-ID: DRKS00004878). Ethical approval was granted by German Sport University Ethics Committee (reference number: 56/12). The study was conducted in compliance with the Helsinki Declaration. The present evaluation was conducted as a cross-sectional study based on baseline data of the RCT [22]. Eligible patients were 18 to 65 years of age and starting an inpatient medical rehabilitation treatment because of LBP. Patients were invited to participate in the study during an information seminar on PA. All patients receiving inpatient rehabilitation because of LBP were included in the study. Exclusion criteria were cognitive disorders, lack of understanding the German language, any kind of surgery within the last three months, posttraumatic conditions (e.g. LBP after an accident) a current state pension claim and refusal of participating in the RCT. At the time of the data collection, patients did not know whether they had been randomized into the intervention or control group. In the period of recruitment 931 LBP patients were eligible to participate in the study. All patients participating signed written informed consent to participate in the RCT. Patient recruitment was conducted monocentric in an inpatient rehabilitation center of the German Statutory Pension Insurance Rhineland. Data were collected consecutively from April 2013 to April 2014.

### Measurements

Patients answered a questionnaire on PA, HRQoL and sociodemographic aspects. HRQoL was measured using the EQ-5D, a standardized, preference based measure of health status. Preference-based approaches are often used in cost-effectiveness analyses and incorporate values or utilities for health outcomes [23]. The EQ-5D is designed for self-completion and assesses five dimensions of HRQoL: mobility, self-care, daily activities, pain and discomfort, anxiety or depression [24]. In the present study, the new version of the questionnaire, EQ-5D-5 L, was applied [25]. EQ-5D-5 L provides five levels for answering each dimension: no problems, slight problems, moderate problems, severe problems and extreme problems. Descriptive answers were converted into a single summary index by applying a formula that essentially attaches weights to each of the levels in each dimension (German version) and using the crosswalk value set for the EQ-5D-5 L. The crosswalk is based on a response mapping approach that estimates the relationship between responses to the EQ-5D-3 L (‘3 L’) and EQ-5D-5 L (‘5 L’) descriptive systems, and subsequently established a link to the established 3 L value sets [25]. Index value of health status range from ‘1’ (perfect health) to ‘0’ (death). States perceived to be worse than death have a negative summary score index.

PA was operationalised by Global Physical Activity Questionnaire (GPAQ) [26,27]. The GPAQ collects information on PA during a typical week within three domains (or settings) as well as sedentary behavior. The domains referred to are PA at work (paid and unpaid), transport activity (travel to and from places, for example by walking and cycling) and recreational activities (e.g., sports, fitness and recreational activities (leisure)). The GPAQ comprises of 16 questions and, for each domain, PA is assessed if it was continuously performed for at least ten minutes. Work and leisure time physical activity are specifically measured with respect to their intensity^a^. Therefore, vigorous intensity is described as “activities that cause large increases in breathing or heart rate”. The GPAQ is scored by multiplying the minutes per week for each domain by their associated MET to create MET-min scores per week. In the comparison with the International Physical Activity Questionnaire (IPAQ), a previously validated and accepted PA questionnaire, the GPAQ showed a moderate to strong positive relationship (concurrent validity: Spearman’s rho 0.45–0.65). Pedometer studies showed fair pooled criterion validity for total PA (r = 0.31) and reliability was of moderate to substantial strength (kappa from 0.67 to 0.73; Spearman's rho from 0.67 to 0.81) [27]. A further study evaluating criterion validity in comparison with accelerometer showed that the minutes of moderate and vigorous PA were correlated with the accelerometer measurements of moderate (*r* = 0.28) and vigorous (*r* = 0.48) PA. GPAQ data were checked for possible data entry errors by using the provided WHO CleanRecode program [28]. Additionally, plausibility check was performed. Based on interdisciplinary discussions, possible maximum for work related PA was defined 2400 min/week (equates to eight hours a day five days a week). For transport leisure time PA, maximum of 3360 min/week (equates to eight hours a day seven days a week) was considered as maximum. Data higher than the defined maximum were set to the defined maximum.

Sociodemographic variables collected were *age, gender, partnership, level of education and body mass index (BMI).* BMI data were classified: BMI from 20 – 24.9 was defined “normal weight”, BMI from 25 – 29.9 “overweight” and ≥ 30 “obese” [29]. Additionally, work-related and indication-specific variables were collected. Work-related variables refer to *current employment status (employed/unemployed), sick certificate before rehabilitation (<6 weeks/≥ 6 weeks), current work ability compared to highest work ability ever (Assume that your work ability at its best has a value of 10 points. How many points would you give your current work ability? (0 means that you currently cannot work at all))* as an item from work ability index [30]. Indication-specific variables were *duration of LBP (≤12 months/> 12 months)* and *intensity of pain.* Intensity of pain during the last four weeks” was measured via a question from the SF-36 questionnaire (“How much bodily pain have you had during the past 4 weeks?”; answering on a scale from 1 (none) to 6 (very severe)) [31].

### Statistical analysis

#### Coding of variables

Physical inactivity was defined as 0 min PA/week, equivalent to not performing PA continuously for at least ten minutes in the life domain referred to in the GPAQ. For the regression models, the PA variables were classified: Transport PA was dichotomized (“no transport PA (0 min/week)”/“transport PA”), workplace PA and leisure time PA were divided into three classes each. Cut-off at workplace PA was set at “no workplace PA (0 min/week)”/“moderate or vigorous workplace PA”/“moderate and vigorous workplace PA”. Classification of leisure time PA was conducted referring to WHO-recommendations (“no leisure time PA (0 MET-min/week)”/“<600 MET-min”/“≥600 MET-min”).

#### Descriptive presentation

In order to describe the sample, means and standard deviation (SD) were calculated for continuous data and frequency tables (n; %) for categorical data. For the description of HRQoL, the frequency tables of self-reported problems across each of the five EQ-5D domains were presented. Summary index value of health status (EQ-5D-5 l) and the volume of PA at the workplace, in transport and in leisure time were described by means, SD, median, first and third quartile. The results on the volume of PA only relate to patients reporting any PA (>0 min PA/week). Prevalence of physical inactivity was presented separately by frequency of patients reporting “no PA” in each life domain.

#### Statistical tests

Gender and age differences between participants in the study and non-participants were tested by Pearson Chi Square Test respectively Mann–Whitney-*U* Test.

To explore the association between PA and HRQoL (question 1) and identify variables associated with HRQoL (question 2) multiple linear regression models were calculated. In the first model (question 1), solely the different domains of PA were included. In the second model (question 2), besides PA, the sociodemographic, work-related and indication-specific variables were included stepwise to get insight in what variables explained HRQoL best. The principal assumptions of linear regression were tested. In both regression models, participants with missing values in dependent or independent variables were excluded. For all statistical tests, significance level was set at p < 0.05. All analyses were run with IBM SPSS Statistics 22.

## Results

### Sample description

Of 931 eligible patients, 412 gave informed consent and completed the questionnaire (participation rate = 44%). Compared to non-participants, there was no significant difference regarding sex (p = 0.18), but regarding age (p = 0.04). In average, participants (mean = 50.4 years; SD = 8.1) were one year younger than non-participants (mean = 51.3 years; SD = 8.2; *t*-test p = 0.04). Most frequent reasons cited for non-participation were *privacy protection* (n = 99; 19%) and *language problems* (n = 91; 18%). 288 non-participants (55%) did not state a reason.

The sample consisted of 286 men (69.4%) and the mean age was 50.4 (±8.1) years. Sociodemographic and indication-specific variables as well as work-related aspects of the sample are shown in Table 1.

**Table 1:** **Sample description**

**Whole population n = 412**	
***Sociodemographic variables***	
**Age** (years) (mean; SD) (n = 406)	50.4 (8.1)
**Gender:** men (n; %) (n = 412)	286 (69.4%)
**BMI** (mean; SD) (n = 383)	29.3 (5.6)
**Overweight** (BMI ≥ 25 - < 30) (n; %)	166 (43.3%)
**Obese** (BMI ≥ 30) (n; %)	144 (37.6%)
**Living in partnership** (n; %) (n = 400)	308 (77.0%)
**Level of education (secondary modern school)** (n; %) (n = 399)	214 (53.6%)
***Work-related aspects***	
**Sickness certificate before rehabilitation > six weeks** (n; %) (n = 396)	148 (37.3%)
**Currently employed** (n; %) (n = 395)	318 (80.5%)
**Job satisfaction during the last four weeks (0 – 7)** (mean; SD) (n = 360)	3.2 (2.2)
**Current workability (0 – 10)** (mean; SD) (n = 387)	4.5 (2.5)
***Indication-specific variables***	
**Duration of LBP > 12 months** (n; %) (n = 400)	343 (85.8%)
**Intensity of pain during the last four weeks (min: 1; max: 6)** (mean; SD) (n = 378)	4.6 (1.0)

### Descriptive results

The prevalence of physical inactivity across different life areas is presented in Table 2. Vigorous leisure time shows highest prevalence of physical inactivity (71%). Independent of intensity, 45% of the patients are not physically active at the workplace. In leisure time, 53% show neither moderate nor vigorous PA. 19% (n = 79) do not show any PA of at least ten minutes duration in total PA, respectively in any life area (see Table 2).

**Table 2:** **Frequency tables of physically inactive patients**

**Life domain and intensity of PA**	**Number of patients reporting no physical activity (n; %)**
**No work PA**	185 (45 %)
No vigorous work PA (n = 412)	251 (61%)
No moderate work PA (n = 412)	255 (62%)
**No transport PA** (n = 412)	238 (58 %)
**No leisure time PA**	218 (53 %)
No vigorous leisure time PA (n = 412)	291 (71 %)
No moderate leisure time PA (n = 412)	278 (68 %)
**No PA in all domains**	79 (19 %)

PA data (min/week) of patients reporting PA are displayed in Table 3. In patients reporting PA, PA level at work is higher than in leisure time and PA for transportation. While median in moderate workplace PA is 840 min/week and in vigorous workplace PA even 1350 min/week, duration of transport (median = 270 min/week) and leisure time PA (vigorous: median = 150 min/week; moderate: median = 138 min/week) is considerably lower.

**Table 3:** **Domain specific physical activity of patients reporting PA**

**Whole population n = 412**	**Mean**	**SD**	**Min; Max**	**Median**	**25%-; 75%-percentile**
**Vigorous work PA **(min/week) (n = 161)	1303	812	20; 2400	1350	495; 2100
**Moderate work PA** (min/week) (n = 157)	1007	787	60; 2400	840	360; 1500
**Transport PA** (min/week) (n = 174)	447	561	20; 3360	270	120; 540
**Vigorous leisure time PA** (min/week) (n = 121)	242	250	20; 1200	150	90; 300
**Moderate leisure time PA** (min/week) (n = 134)	244	346	20; 3360	138	90; 270

Table 4 shows frequency tables from the five domains of the EQ-5D instrument as well as mean index value of health status (dependent variable). Descriptive results from the five domains of the EQ-5D instrument showed most frequent severe (25.5%) respectively extreme problems (8.9%) in *pain and discomfort*, followed by *daily activities* (severe: 20.8%; extreme: 2.2). Furthermore, severe problems in *mobility* are answered by 11.2%. Majority of the patients reported no problems in *self-care* (57.3%). The mean index value of health status (HRQoL) was 0.65 (±0.22).

**Table 4:** **Health Related Quality of Life**

**Whole population n = 412**	**No problems n (%)**	**Slight problems n (%)**	**Moderate problems n (%)**	**Severe problems n (%)**	**Extreme problems n (%)**
**Mobility** (n = 366)	81 (22.1%)	97 (26.5%)	145 (39.6%)	41 (11.2%)	2 (0.5%)
**Self-care** (n = 372)	213 (57.3%)	91 (24.5%)	63 (16.9%)	4 (1.1%)	1 (0.3%)
**Daily activities** (n = 371)	34 (9.2%)	105 (28.3%)	147 (39.6%)	77 (20.8%)	8 (2.2%)
**Pain and discomfort** (n = 361)	9 (2.5%)	55 (15.2%)	173 (47.9%)	92 (25.5%)	32 (8.9%)
**Anxiety or depression** (n = 351)	118 (33.1%)	108 (30.3%)	90 (25.2%)	29 (8.1%)	12 (3.4%)
	**Mean**	**SD**	**Min; Max**	**Median**	**25%; 75%-percentile**
**Index value of health status (0 – 1)** (n = 319)	0.65	0.22	−0.11; 1.00	0.72	0.49; 0.81

For calculation of regression models, independent variables were classified as described in statistical analysis. Table 5 shows frequency tables of PA classes.

**Table 5:** **PA classes (independent variables in the regression models)**

	**N (%)**
**Workplace PA** (n = 412)	
“No workplace PA”	185 (44.9%)
“Moderate or vigorous workplace PA”	136 (33.0%)
“Moderate and vigorous workplace PA”	91 (22.1%)
**Transport PA** (n = 412)	
“No transport PA”	238 (57.8%)
“Transport PA”	174 (42.2%)
**Leisure time PA** (n = 412)	
“No leisure time PA”	218 (52.9%)
“Leisure time PA < 600 MET-Min/week”	55 (13.3%)
“Leisure time PA ≥ 600 MET-Min/week”	139 (33.7%)

In the first step, model 1, solely including dimensions of PA, was calculated (Table 6). There was a significant negative association of “moderate and vigorous workplace PA” and HRQoL (β = −0.064; p = 0.04). Patients reporting “moderate and vigorous workplace PA” were more likely to report lower HRQoL compared to patients reporting “no workplace PA”. Furthermore, patients achieving WHO recommendations in leisure time (≥600 MET-min/week) showed a significantly higher HRQoL compared to patients reporting “no leisure time PA” (β = 0.068; p = 0.01). Overall, PA explained 3% of the variance of HRQoL (R^2^ = .033).

**Table 6:** **Model 1 – Association of PA and Health Related Quality of Life**

**N = 319**	**Beta**	**SE (β)**	**95%-CI**	**Sig.**
**Workplace PA**				
“Either moderate or vigorous workplace PA” vs. “no workplace PA”	−0.032	0.029	−0.089; 0.025	0.27
”Moderate and vigorous workplace PA” vs. “no workplace PA”	−0.064	0.032	−0.126; −0.002	0.04*
**Transport PA**				
“Yes” vs. “no”	0.002	0.025	−0.047; 0.052	0.92
**Leisure time PA**				
“<600 MET-min/week” vs. “no leisure time PA”	0.068	0.039	−0.008; 0.144	0.08
“≥600 MET-min/week” vs. “no leisure time PA”	0.068	0.028	0.014; 0.123	0.01*

Dependent variable: summary health index (EQ-5D); R^2^ = .033; *p < 0.05; overall p = 0.06.

In model 2 (see Table 7) only two independent variables were statistically significant: The higher the current work ability compared to highest work ability ever, the higher was HRQoL (β = 0.030; p < 0.01). Furthermore, intensity of pain during the last four weeks was negatively associated with HRQoL, meaning that higher intensity of pain goes in line with lower HRQoL (β = −0.104; p <0.01). These two variables explained 35% of the variation in HRQoL (R^2^ = 0.350). In this regression model, no domain of PA showed a significant association.

**Table 7:** **Model 2 – variables associated with Health Related Quality of Life**

**N = 267**	**Beta**	**SE**	**95%-CI**	**Sig.**
**Intensity of pain during the last four weeks (min: 1; max: 6)**	−0.104	0.013	−0.130; −0.078	<0.01*
**Current work ability (min: 0; max: 10)**	0.030	0.005	0.020; 0.040	<0.01*

Dependent variable: summary health index (EQ-5D); R^2^ = 0.350; *< 0.05; overall p <0.01.

Excluded variables: BMI, current employment status, sick certificate before rehabilitation, duration of LBP, workplace PA, leisure time PA, transport PA, age, gender, partnership, level of education.

## Discussion

Main finding of the present study was that “moderate and vigorous workplace PA” showed a negative association with HRQoL while patients achieving WHO recommendations in leisure time showed a significantly higher HRQoL compared to patients reporting “no leisure time PA”. Hence, solely the variables “current work ability” and “intensity of pain” contributed significantly to explain variance in HRQoL.

First, the descriptive data of PA are discussed from an application-oriented point of view in order to identify whether lack of PA is a lifestyle factor to be targeted in this sample. The prevalence of persons achieving WHO recommendations corresponded to the results of representative surveys in Germany. There, different surveys showed a prevalence from 20.5% [32] to 54% [33] what compares favorably with the results in the present study. The discussion, whether persons with CLBP show lower levels of PA is controversial. According to Lin et al. [34], chronic LBP patients with high levels of disability are likely to have low levels of PA. Several other studies showed that the mean activity of patients with chronic LBP does not differ from healthy individuals [35-37].

The present evaluation focused on the evaluation of PA, as an important lifestyle factor, and its association to HRQoL. By investigating the associations of PA and HRQoL in LBP patients, especially the different types of PA should be taken into account. Regarding the domain specific minutes per week reported in the present study, the high values of workplace PA compared to leisure time PA were eye-catching. Scientific literature shows, that the differentiation of workplace and leisure time PA as well as intensity of PA is relevant in the context of assessing potential health-enhancing effects of PA in LBP. Our regression on the associations of PA and HRQoL (model) supported the assumption of opposed effects of leisure time PA and workplace PA: While “moderate and vigorous workplace PA” showed a negative association (β = −0.064; p = 0.04), leisure time PA “≥600 MET-min/week” showed a positive association (β = 0.068; p = 0.01) to HRQoL. Independently of HRQoL, Jacob et al. showed that strenuous PA at work might lead to a significantly higher risk of back pain [38]. Junqueira and colleagues [39] showed higher prevalence of LBP associated with heavy work while moderate activities such as jogging seemed to be beneficial. Furthermore, there is evidence that the exposure to awkward positions is a risk factor for LBP. This is an aspect that was not measured in the present study and therefore needs to be considered in further investigations. Also Schneider and Schiltenwolf [40] found that LBP prevalence in occupations associated with high intensity and high volume PA at work was higher-than the average. Moreover, they show that professional groups associated with light PA, e.g. senior management, showed a lower prevalence [40]. Study results concerning leisure time PA, sports, physical exercise and risk of LBP are inconsistent: intensive activities are considered a risk factor for LBP, whereas moderate PA is, on the contrary, considered beneficial to health [41].

However, the findings showed no significant association of PA and HRQoL if further variables were considered to explain influences on HRQoL. The results contradict other studies, stating a relationship between level of PA and quality of life in LBP patients [42] as well as in general population [10,43]. Only two of the 14 variables included significantly contributed to explain the variance of HRQoL. As expected, high intensity of pain was related to low HRQoL (β = −0.104; p < 0.01) what emphasizes the importance of pain management in CLBP patients. The association of current work ability (β = 0.030; p < 0.01) added weight to the assumption that especially workplace plays a crucial role regarding HRQoL. This is in line with results from Sörensen et al. [44] that suggest that the promotion of work ability may have beneficial effects on the quality of life. In consequence, rehabilitation practitioners and future research should focus on work-related aspects and its associations to HRQoL and in this context emphasize employment and vocational orientation in medical rehabilitation.

This research investigation has several strengths and limitations. As inclusion criteria were defined very broad, all patients prescribed inpatient rehabilitation because of LBP were included in the study. Due to logistic restrictions, specific problems related to LBP (e.g., leg pain; acute pain) could not be obtained. Also the participation rate of 44% and the monocentric study design are a limitation as they may cause a selection bias. Furthermore, as the present evaluation was performed in the context of an intervention study on promoting health-enhancing PA, primarily motivated patients may have responded. Due to missing values in independent variables, the regression models could only be fitted on subpopulations and therefore a further selection bias cannot be excluded. In consequence, implications for population mean of LBP patients have to be concluded very carefully. A second limitation is that the cross-sectional data cannot elucidate a possible causality of the associations. Thirdly, the self-reported operationalization of PA may induce a reporting bias due to overestimation [45]. To minimize this potential bias, a conservative plausibility data check was performed as described in the chapter methods. Hence, the decision about plausibility was only made on the basis of interdisciplinary discussions between practitioners and researchers and therefore no reference on cut-off values can be provided.

Apart from that, this study includes a number of important strengths. One such strength is the domain specific measurement of PA. By examining PA in different life areas and evaluating its domain specific associations to HRQoL, a gainful contribution assessing health-enhancing effects of PA in LBP is made. Furthermore, the comprehensive data collection enabled to capture often overlooked information such as work-related aspects. Another strength of the present study certainly is its concentration on HRQoL as an highly important outcome in rehabilitation and health services research. Whereas several studies and reviews on the relationship of PA and LBP are available, there is a lack of studies evaluating the association of PA and HRQoL in LBP patients. The present study therefore encounters the importance of the construct of HRQoL as a superior aim in rehabilitation and heath care service, as emphasized in German treaties on social law and utility analysis [8].

## Conclusion

Leading patients to an active lifestyle is a superior aim of exercise therapy in rehabilitation [19,46]. Thereby, greater weight should be given to the life domain in which PA is conducted and especially leisure time PA should be promoted as it was associated with higher HRQoL. However, interventions on the promotion of health-enhancing PA should consider volume and intensity of workplace PA (e.g., consulting on how to reduce health-related PA load in patients with high workplace PA) and psycho-social aspects.

In the context of HRQoL it must be assumed that the relevance of PA is overestimated. However, as HRQoL is of utmost importance in rehabilitation and health care research, further research should be performed on the prognostic influence of PA and other relevant predictors on HRQoL in longitudinal studies. Thereby, particular attention should be paid to the patients’ work-related aspects.

## Endnote

^a^The metabolic equivalent (MET) is a physiological measure expressing the expended energy of physical activities. MET is defined as the ratio of the rate of energy consumption during a specific physical activity to a reference metabolic rate. Vigorous intensity corresponded to 8 MET; moderate intensity corresponded to 4 MET.
